# Supplementary material for: Pulse wave velocity is a new predictor of acute kidney injury development after off-pump coronary artery bypass grafting
Source: PLoS One. 2020 Apr 30;15(4):e0232377. doi: 10.1371/journal.pone.0232377 (PMC7192459; doi:10.1371/journal.pone.0232377)
Supplement: S1 Table — (DOCX) [file pone.0232377.s001.docx]

|  | **Low baPWV (n = 118)** | **High baPWV (n = 46)** | ***p*-value** |
| --- | --- | --- | --- |
| Female, n (%) | 27 (23) | 21 (46) | 0.004 |
| Age, years | 64.0±10.5 | 72.0±7.2 | < 0.001 |
| Age > 75 years, n (%) | 12 (10) | 15 (33) | < 0.001 |
| Body mass index, kg/m^2^ | 24.3±3.2 | 23.9±3.3 | 0.469 |
| Obesity, n (%) | 55 (47) | 18 (39) | 0.387 |
| Hypertension, n (%) | 79 (67) | 38 (83) | 0.046 |
| Diabetes, n (%) | 49 (42) | 38 (83) | < 0.001 |
| *under insulin therapy, n (%)* | 9 (8) | 15 (33) | < 0.001 |
| Dyslipidemia, n (%) | 35 (30) | 19 (41) | 0.154 |
| History of cerebrovascular accidents, n (%) | 17 (14) | 9 (20) | 0.416 |
| Peripheral arteriopathy, n (%) | 29 (25) | 16 (35) | 0.188 |
| Chronic obstructive pulmonary disease, n (%) | 7 (6) | 3 (7) | 1.000 |
| Unstable angina, n (%) | 72 (61) | 32 (70) | 0.307 |
| Acute MI, n (%) | 17 (14) | 7 (15) | 0.561 |
| Recent MI, n (%) | 12 (10) | 3 (7) | 0.596 |
| Old MI, n (%) | 7 (6) | 1 (2) | 0.444 |
| Single-vessel disease, n (%) | 5 (4) | 2 (4) | 1.000 |
| Double-vessel disease, n (%) | 16 (14) | 10 (22) | 0.198 |
| Triple-vessel disease, n (%) | 97 (82) | 34 (74) | 0.234 |
| Left main disease, n (%) | 40 (34) | 16 (35) | 0.915 |
| History of coronary intervention, n (%) | 15 (13) | 7 (15) | 0.672 |
| Atrial fibrillation, n (%) | 1 (1) | 1 (2) | 0.484 |
| Left ventricle ejection fraction (%) | 55.6±13.8 | 61.4±8.5 | 0.002 |
| Creatinine, mg/dL | 0.9 (0.5-10.8) | 1.0 (0.5-6.2) | 0.492 |
| MDRD-GFR, mL/min/1.73 m^2^ | 86.1 (5.1-155.2) | 75.7 (6.9-118.0) | 0.019 |
| Chronic kidney disease stage ≥ 4 | 12 (10) | 8 (17) | 0.204 |
| Mean baPWV, m/s | 15.1±2.3 | 22.4±5.3 | < 0.001 |
| *right baPWV, m/s* | 15.1±2.7 | 22.0±3.8 | < 0.001 |
| *left baPWV, m/s* | 15.0±2.4 | 22.8±7.2 | < 0.001 |
| EuroSCORE II | 1.3 (0.5-21.4) | 1.9 (0.8-6.9) | 0.001 |
| AKI, acute kidney injury; baPWV, brachial-ankle pulse wave velocity; GFR, glomerular filtration rate; MDRD, Modification of Diet in the Renal Disease; MI, myocardial infarction. | | | |

**S1 Table. Comparison of baseline characteristics between low and high baPWV groups.**
