# Supplementary material for: Pulse wave velocity is a new predictor of acute kidney injury development after off-pump coronary artery bypass grafting
Source: PLoS One. 2020 Apr 30;15(4):e0232377. doi: 10.1371/journal.pone.0232377 (PMC7192459; doi:10.1371/journal.pone.0232377)
Supplement: Attachment — Submitted filename: Response to Reviewers.docx [file pone.0232377.s002.docx]

**Answers to reviewers’ comments**

Dear Professor Laura Pasin,

We, the authors of this study, have read and discussed the comments that you and the reviewers made. We think that the comments helped improve the quality of this work. **For the matter of our study design** pointed out by you and Reviewer #2, in fact, this study has both prospective and retrospective elements. It is true that the PWV data had been prospectively collected before the operations. On the other hand, some of these data had been already collected for some other study purposes in the cardiology department before surgical consultations were needed, and all of the data existed at the time our protocol was submitted to the IRB for initial approval. In other words, we retrospectively analysed the prospectively collected data. Considering the opinion of the editor and reviewer, we decided to remove the phrase, “In our retrospective study”, from the method section of the Abstract and to replace it with **“… baPWV data that were prospectively collected from 164 patients …”**. The fact that retrospective analysis was performed was expressed indirectly in the Methods section of the main body: **“informed consent was waived** **because this retrospective review of medical records could not adversely affect the rights or welfare of the subjects.”**

You and Reviewer #3 requested a clear **explanation for what baPWV adds to current methods to predict the risk of AKI.** We think the request was based on the rational expectation that a high baPWV would be associated with some additional risk factors contributing to AKI. Although we scoured the literature, we did not find a better explanation than what we had already described in the Discussion section. In a nutshell, the additional risk of AKI associated with high baPWV, for now, is associated with an increased afferent arteriolar tone and an elevated left ventricular filling pressure. The speculated mechanisms, in our opinion, are clearly described with the references in the Discussion section **(page 15, line # 288 – page 16, line # 302).**

Additionally, we should mention that **the mechanical ventilatory support durations were longer among patients with high baPWVs.** Because these duration data were not normally distributed, we changed the statistical methods to incorporate the use the Mann–Whiney test instead of t-tests, which led to statistical significance. This fact is now described in the Results section and in the abstract. **This is the only statistical consideration that has changed after the revision.**

**For Reviewer #1’s comments**, **firstly**, our primary endpoint was investigating whether or not postoperative development of AKI was associated with preoperative baPWV and then how much and how independently they were associated. We did specify when this study was conducted with the use of “preoperatively” in the first sentence of the subsection, “Measurement of brachial-ankle pluse wave velocity”: **“baPWV measurements were preoperatively performed using a volume-plethysmographic apparatus... .“** **Secondly**, it is right that PWV can be influenced by various factors like inotropic drugs and patients’ hemodynamic status postoperatively, but the PWV measurements that were dealt with in this study were all done preoperatively. However, even preoperative PWV might be influenced by what kind of medications patients are taking. So, we modified the sentence in the subsection, “Study limitations”: **“antihypertensive medications, such as vasodilators that the patients were taking before surgery, might have influenced the baPWV measurements, but they were not controlled.”** **Thirdly,** the baPWV calculation method was described in detail in the subsection, “Measurement of brachial-ankle pulse wave velocity”: **“To calculate the baPWV, the virtual length of the arterial path between the heart and the brachial artery (Lhb) and the length of the path between the heart and the posterior tibial artery (Lha) should be derived from the subject’s height using the appropriate formulas. Then, the time difference between the beginning points of systolic increase in brachial and ankle pressure waves (Tba) should be measured. Finally, baPWV was calculated by the path length difference devided by the time difference (baPWV = (Lha – Lhb)/Tba).”** **Fourthly,** we separated the operation data (Table 2) from the baseline data (Table 1), and, with that, some more important data were added to the respective Tables. **Lastly,** Figures 1 and 2 have been modified for clarity.

**For Reviewer #2’s comments**, **firstly**, the changes in the text we made regarding the study design have already described in the letter to the editor. We can say that this work involved a retrospective analysis of prospectively collected data. **Secondly,** we have added a patient selection flowchart in Fig 1. **Thirdly,** as Reviewer #2 pointed out, creatinine level, GFR, and EuroSCORE II were not normally distributed. So, these data have been presented as median (range) instead of mean values, and the comparison was performed by a nonparametric method, the Mann-Whitney *U* test. These results have been presented in Table 1. Of course, the statistical results have not been changed. The relevant text has also been corrected in accordance with these results in the statistical subsection and in the subsection, “Association of baseline characteristics with postoperative AKI” We did the same thing with some other data that were not normally distributed. With this, more surgical data about the off-pump CABG were added and reported in a separate table (Table 2). Besides, the surgical technique used during the study period was introduced in the subsection, *Surgical techniques*. **Fourthly**, we provided the positive and negative predictive values of high baPWV for AKI and compared these with those of creatinine. This is described in the subsection*,* “Independent predictors of postoperative AKI”**: “Moreover, using preoperative baPWV and serum creatinine cut-off values of 18.2 m/s and 1.0 mg/dL, respectively, from the respective ROC curves, the positive and negative predictive values for baPWV are 43.5% and 91.5%, respectively, compared with 32.7% and 88.4%, for creatinine. Therefore, baPWV is likely to be more useful in terms of predicting postoperative AKI.”** **Lastly,** this study had some elements of retrospective design with their own limitations. We think that these limitations have already been mentioned. The whole text was thoroughly reviewed by a native English speaker with expertise in this field.

**For Reviewer #3’s comments**, first of all, we appreciate that the reviewer positively touched on every detail to improve this manuscript. **For comment #1,** we presented additional baseline data of the patients with high and low baPWV in a supplementary appendix, for reference. This supplementary table was specified in the subsection, “Impact of high baPWV on postoperative outcomes”: **“…despite the fact that preoperative creatinine levels of the high PWV group was not statistically different from those of the low PWV group, and preoperative median eGFR value of the low PWV group was 75.7 mL/min/1.73 m^2^ which was not bad. For further details about comparison of baseline characteristics between the high and the low baPWV groups, please see supplementary material (S1 Table).”** **For comment #2,** we also wanted to see the correlation between the high baPWV and the development of more advanced AKI, but there were no instances of stage 3 AKI or renal replacement therapy in this patient cohort. Even the incidence of stage 2 AKI was insufficient to perform a valid analysis. Anyway, postoperative AKI data were stratified by stage and presented in the Table 4. **For comment #3,** we specified follow-up details in the subsection, “Patient selection”: **“The median follow-up duration was 39.2 months (range, 1.6 - 78.0 months).** **There was no loss to follow-up among the included patients.”** Late mortality was as follows: **“Late mortality was defined as any-cause mortality after POD 30”** in the subsection, “Renal function and postoperative complications.” **For comments #4 & #5,** like the results of some studies dealing with the impact of postoperative AKI on long-term renal function, we expect there would be a long-term correlation between renal function and baPWV even in this cohort. For example, Ryden et al.^*^ found that even a small increase in serum creatinine after CABG was associated with a 3-fold increased risk of ESRD development in the long-term. But we think that this is beyond the scope of our study focusing on postoperative AKI. *(^*^Rydén L, Sartipy U, Evans M, Holzmann MJ. Acute Kidney Injury After Coronary Artery Bypass Grafting and Long-Term Risk of End-Stage Renal Disease. Circulation. 2014;130(23):2005-11).* **For comment #6,** we think that many things about the borderline inter-group difference of the composite neurologic outcome composed of stroke and delirium, have already been covered in the Discussion section, including an acknowledgement of the borderline significance, a brief introduction of recent relevant publications, and the necessity for additional studies to elucidate this correlation. Therefore, do you really think we still need to place the same acknowledgement again in the limitations subsection? **For comment #7,** concerning this selection bias, we never had PWV measurements skipped only because patient risk was very low. Some of the measurements were not performed because it was just not a routine preoperative evaluation during the early period, some measurements were refused by patients due to the fact that the cost was not being covered by insurance, and some were skipped in urgent or emergency contexts. This is briefly mentioned in the limitations subsection. This is one of the typical limitations of retrospective studies that do not include prospective controls. **For comment #8,** the relatively long median durations of ICU and hospital admissions in our study were mainly due to the inclusion of several outliers. Because these duration data were not normally distributed, we compared the median values rather than mean values using the Mann-Whitney non-parametric test, showing a little shorter duration (ICU stay and hospital stay, 1.7 and 9 days, respectively, in low baPWV group vs. 1.9 and 9 days in high baPWV group, *p* = ns). This has been presented in Table 4. However, you may still think these durations looks a bit long. We think this was due to several factors, like manpower shortages, patients’ extreme reluctance to be discharged early, rare pressure from the management of our city government hospital. For example, the vast majority of the patients undergoing cardiac surgery do not move to a general ward on the day of surgery, regardless of how fast the extubation is done and how good the patient’s condition is. **For comment #9,** postoperative pneumonia is now defined as follows: **“Postoperative pneumonia was defined as a lower respiratory tract infection with accompanying consolidation detected on chest x-ray,”** in the subsection, “Renal function and postoperative complications.” **For comment #10,** PMI (perioperative myocardial infarciton) was not diagnosed only with troponin level, but with considering 3 factors: CK-MB, troponin I, and EKG findings. We have already described this in the subsection, “Renal function and postoperative complications”**: “Perioperative myocardial infarction was defined as creatine kinase (CK-MB) concentration >40 ng/mL, new Q waves noted on electrocardiogram, or peak troponin I levels >15 ng/mL at 12 hours after operation.”** **For comment #11,** this entire manuscript was thoroughly reviewed by a native English speaker with expertise in this field.

Sincerely Yours,

*Jae-Sung Choi, MD, PhD,*

Clinical professor, SNU-SMG Boramae Medical Center, Seoul, Korea

*Jeong Sang Lee, MD, PhD*

Professor, Dept. of Thoracic & Cardiovascular Surgery, Seoul National University College of

Medicine, SNU-SMG Boramae Medical Center, Seoul, Korea
