# Supplementary material for: Pulse wave velocity is a new predictor of acute kidney injury development after off-pump coronary artery bypass grafting
Source: PLoS One. 2020 Apr 30;15(4):e0232377. doi: 10.1371/journal.pone.0232377 (PMC7192459; doi:10.1371/journal.pone.0232377)
Supplement: Attachment — Submitted filename: Response to Reviewers(second revision).docx [file pone.0232377.s003.docx]

**Answers to reviewers’ comments**

Dear Professor Laura Pasin,

We, the authors of this study, have read and discussed the new comments from the reviewer #3 and we made a minor revision.

**For the issue of sample size calculation,** because this study was neither a typical prospective nor a randomized controlled trial, we did not predetermine a sample size. As the reviewer #3 knows well, unlike prospective randomized controlled studies, retrospective review studies usually use statistical power rather than the calculation of sample sizes, thus, we performed the post hoc power analysis. The power (1-β) that we calculated was 99.3% for the statistical comparison of AKI incidences between the low baPWV group and high baPWV group, and 84.0% for the comparison of mean baPWV between the AKI group and no-AKI group. By the way, although the powers are fairly acceptable, we don’t think that reporting this fact is necessary because the observed powers (or post-hoc) are directly related to the p-values that were already demonstrated as < 0.001 in our study. In short, we don’t need to distinguish between true negative and false negative with a post-hoc power for our positive study results.

**For the asking to specify that no postoperative RRT event was observed,** we presented this fact in the subsection, “ Impact of high baPWV on postoperative outcomes”: **“There was no patient who required renal replacement therapy in patients with postoperative AKI though.”**

**For the ambiguous definition of PMI,** we redescribed it as, **“Perioperative myocardial infarction was defined based upon an elevation of biomarkers (either creatine kinase (CK-MB) concentration >40 ng/mL or peak troponin I levels >15 ng/mL at 12 hours after operation) and the presence of new pathological Q waves or left bundle branch block.”**

**For the lack of data on long-term relationship between renal function and baPWV,** we add this fact to the limitation subsection as**, “Lastly, we expect there would be a long-term correlation between renal function and baPWV in this cohort, but we could not investigate that because the related data were not available.”**

Sincerely Yours,

*Jae-Sung Choi, MD, PhD,*

Clinical professor, SNU-SMG Boramae Medical Center, Seoul, Korea

*Jeong Sang Lee, MD, PhD*

Professor, Dept. of Thoracic & Cardiovascular Surgery, Seoul National University College of

Medicine, SNU-SMG Boramae Medical Center, Seoul, Korea
